# Biomarkers of lymph node metastasis in esophageal cancer

**DOI:** 10.3389/fimmu.2024.1457612

**Published:** 2024-09-27

**Authors:** Ningzi Wu, Junlan Cai, Junfei Jiang, Ye Lin, Xiaoqing Wang, Weiguang Zhang, Mingqiang Kang, Peipei Zhang

**Affiliations:** Department of Thoracic Surgery, Fujian Medical University Union Hospital, Fuzhou, China

**Keywords:** lymph node metastasis, esophageal cancer, molecular mechanism, biomarker, diagnosis

## Abstract

Esophageal cancer (EC) is among the most aggressive malignancies, ranking as the seventh most prevalent malignant tumor worldwide. Lymph node metastasis (LNM) indicates localized spread of cancer and often correlates with a poorer prognosis, emphasizing the necessity for neoadjuvant systemic therapy before surgery. However, accurate identification of LNM in EC presents challenges due to the lack of satisfactory diagnostic techniques. Imaging techniques, including ultrasound and computerized tomography scans, have low sensitivity and accuracy in assessing LNM. Additionally, the existing serological detection lacks precise biomarkers. The intricate and not fully understood molecular processes involved in LNM of EC contribute to current detective limitations. Recent research has shown potential in using various molecules, circulating tumor cells (CTCs), and changes in the microbiota to identify LNM in individuals with EC. Through summarizing potential biomarkers associated with LNM in EC and organizing the underlying mechanisms involved, this review aims to provide insights that facilitate biomarker development, enhance our understanding of the underlying mechanisms, and ultimately address the diagnostic challenges of LNM in clinical practice.

## 1 Introduction

Esophageal cancer (EC) is the seventh most prevalent cancer and the sixth highest contributor to cancer-related mortality globally (1). Esophageal squamous cell carcinoma (ESCC) accounts for approximately 90% of global EC cases, with high incidence in East Africa, sub-Saharan Africa, and much of Central Asia. In contrast, esophageal adenocarcinoma (EAC) is more prevalent in Europe and high-income North America, where its incidence has quadrupled over the past four decades (2, 3). As immune organs, lymph nodes (LNs) defend against pathogen dissemination and regulate immune responses and homeostasis. Nevertheless, LNs and their lymphatic circulation can serve as conduits for disease progression, contributing to the spread of inflammation and tumors (4). Lymph node metastasis (LNM) is the primary pathway for the spread of most solid tumors. In EAC, LNM increases from less than 5% for intramucosal (pT1a) lesions to 26% for submucosa (pT1b) lesions. For pT1a ESCC, the risk is approximately 4%, while for pT1b, it rises to around 30%, which is higher than in EAC (3).

The esophagus anatomically includes cervical, thoracic, and abdominal segments, with a wide distribution of regional LN drainage and the heterogeneity of LNM patterns in each anatomical region (5). Although tumor cells preferentially metastasize to the corresponding lymphatic drainage area, skip metastasis increases the complexity of LNM in EC (6, 7). The occurrence of LNM in EC is associated with shorter overall survival (OS). A study reported that the 5-year OS rate was over 50% for patients with fewer than one positive LN, and below 30% for those with more than two positive LNs (8). To obtain better clinical outcomes, EC patients with LNM receive lymphatic node dissection and neoadjuvant chemoradiotherapy (NCRT). However, both excessive and insufficient lymph node dissection can harm EC patients because of operative complications, shorter survival, and lower life quality (9, 10). Currently, classification standards of LNM worldwide, clinical practice, and management vary and rely on subjective and personal experience (11, 12). It is essential to accurately determine the presence or absence of LNM and evaluate the extent of metastasis. These assessments facilitate the selection of surgical methods and help evaluate postoperative patient quality and survival.

The imaging modalities include ultrasound, computed tomography (CT), magnetic resonance imaging (MRI), positron emission tomography (PET), and PET/CT, which diagnose LNM based on the size, morphology, and imaging characteristics of the nodes. However, the accuracy of diagnosing cancers and many other diseases relies on the expertise of individual radiologists and pathologists, leading to various comprehension and interpretation of medical images (13). Therefore, current imaging and pathological practices prioritize diagnosing LNM rather than assessing its occurrence, progression, and prognostic value. Biomarkers are measurable and quantifiable characteristics that reflect the physiological and pathological states of the body. Tumor-associated biomarkers are among the essential fields in this area, with extensive research and applications in early diagnosis, progression and prognosis assessment, tumor classification, prediction of treatment responses, and monitoring for recurrence. Traditional tumor markers primarily include proteins secreted by tumor cells and carbohydrate antigens (14, 15). Recent advancements in diagnostic technologies have introduced potential tumor markers, such as genetic mutations, abnormal RNA, epigenetic modifications, circulating tumor cells (CTCs), and microbiome characteristics. These biomarkers help optimize decision-making in clinical practice, particularly in precision oncology, where identifying patients with specific cancer genetic mutations is required for targeted therapy (16, 17).

Consecutive stages of EC progression, from the precancerous, tumor-occurring to premetastatic niche (PMN) stages, are characterized by elements such as hypoxia, acidosis, and changes in the extracellular matrix (ECM), cytokines, inflammatory factors, and tumor-promoting cells that assist in immune avoidance and tolerance. Characteristic products in each stage can be promising biomarkers in LNM of EC. For example, EC cells could secrete IGF2 and elevate VEGF produced by tumor-associated fibroblasts via miR-29c in a p53-dependent manner, activating VEGFR1-positive bone marrow vascular progenitor cells in the tumor microenvironment (TME) and forming PMN (18). After irradiation, the upregulation of MiR-26b-5p in small extracellular vesicles (EVs) derived from dying ESCC cells promotes the proliferation and activation of myeloid-derived suppressor cells and macrophages. These cells suppress the PI3K/AKT pathway by targeting PTEN and eventually facilitate PMN formation (19). Therefore, the primary tumor-derived cells support PMN formation in LNs to aid the spread and colonization of cancer cells. Accordingly, several molecules are promising biomarkers for diagnosing, evaluating, and predicting LNM in EC and other cancer types. The molecular mechanisms underlying LNM are diverse, especially the formation of PMN, which promotes LNM in the esophagus. However, such a microenvironment is complicated, and the specific mechanisms and interactions between molecules and their crosstalk with EC remain insufficient understandings (20). Researchers have focused on the microbiota (21) in the LNM microenvironment, which are essential promoters or regulators of tumor progression.

In this review, we have summarized potential biomarkers that may serve as indicators of LNM in EC and outlined their involvement in the mechanisms, including molecular signaling pathways. This information is expected to guide future biomarker research, deepen researchers’ understanding of the mechanisms underlying LNM in EC, and ultimately offer new tools for clinical practice in the diagnosis and treatment of EC.

## 2 Molecular biomarkers

### 2.1 Proteins and mRNAs

#### 2.1.1 NNMT

NNMT is an important enzyme that controls NAD+ and SAM levels that regulates cellular metabolism, impacting tumor malignancy (22–25). NNMT overexpression has been observed in various cancers (26). Upregulation of NNMT maintains the cancer-associated fibroblast phenotype, activates the PRDM5/COL1A1 axis, and promotes tumor metastasis in gastric cancer (27, 28) and breast cancer (BC) (29). Its inhibitor has been shown to reverse drug resistance in lung cancer cells (30). Therefore, NNMT plays a crucial indicator in cancer progression and offers potential as an effective target for anticancer treatment. NNMT overexpression plays a role in the diagnosis and prognosis of patients with positive LNs through the m6A modification and the EMT pathway (31). SMYD3 expression and the coexpression of histone methyltransferase G9a and MCM7 predict LNM and other adverse outcomes in ESCC (32, 33). Meanwhile, differential methylation of HOXB2 and SEPT9 serves as predictive factors for LNM (34), yet the hypermethylated promoter of FOXF2 only indicates OS in ESCC (35).

#### 2.1.2 MACC1

MACC1, prevalent in various solid tumors, has emerged as a predictor in tumor stages. Its overexpression is linked to diminished OS in tumors and decreased metastasis-free survival in colorectal cancer (CRC) (36, 37). In CRC and BC, MACC1 overexpression regulates β-catenin, HGF/c-Met, and other signaling pathways to elevate tumor malignancy (37, 38). Its overexpression is closely related to LNM in nasopharyngeal and gastric cancer (39, 40). This understanding guides ongoing research into MACC1-based therapeutic strategies to inhibit tumor growth and metastasis. MACC1 overexpression in EAC has been identified as a predictor of metastasis and other poor survival via the MEK1/ERK signaling pathway both *in vitro* and *in vivo* (41). In ESCC, coexpression of MACC1 with Snail and AGR2, along with diminished KAI1, is associated with LNM in ESCC and head and neck squamous cell carcinoma (HNSCC) (42, 43). MACC1 overexpression promotes the LNM via the c-Met/cyclin D1, the PTEN/PI3K/Akt, and the AMPK/ULK1 signaling pathways (44, 45). Moreover, MACC1-AS1, induced by NSD2, mediates cisplatin resistance in ESCC and represents a promising target for enhancing cisplatin-based chemotherapy (46). Therefore, MACC1, with multiple regulatory functions, is identified as a potent indicator and therapeutic target in LNM of EC.

#### 2.1.3 MMPs and TWIST1

MMPs are enzymes responsible for degrading the collagen and other proteins in the ECM (47), and TWIST1 is an essential EMT-inducing transcription factor (48). The upregulation of crosstalk between MMPs and TWIST1 with other markers, such as HLAG-1 (49) and CD44 (50), indicates the occurrence and development of tumor metastasis. A significant inverse correlation between INPP5A and the overexpression of TWIST1, EGFR, and MMP-2 has been observed, which might predict a poor prognosis in ESCC (51). Meanwhile, upregulation of CD147 and MMP-9 could potentially predict LNM and the prognosis in EAC (52).

TWIST1 also affects lymphatic status by upregulating Notch signaling genes (53). Meanwhile, MAML1 is a transcriptional co-activator in the Notch signaling pathway to promote tumor progression and metastasis. Studies have demonstrated that coexpression of TWIST1 and MAML1 in this pathway promotes a cascade in the aggressiveness and metastasis of HNSCC and ESCC (54, 55). However, in another study, no significant changes in MAML1 expression has been found when TWIST1 overexpresses (53). Further studies are required to explore the molecular mechanisms of TWIST1 and MAML1 and other Notch signaling genes.

ADAMs enhance structural resemblance by exhibiting MMP activity. ADAM17 overexpression is linked to LNM and a poorer prognosis in ESCC, contributing to the formation of lymphatic tubules (56). Cancer-associated fibroblasts stimulate ADAM17 through activation of the ERK1/2 pathway to promote the progression of ESCC (57). Its antibody shows promise as a therapeutic target by inhibiting both EGFR-mediated and non-EGFR-mediated pathways to suppress tumor growth (58). ADAM10 overexpression has been demonstrated as a predictor of LNM in oral SCC (59). CLCA4 involves MMP activity and intracellular calcium-gated chloride channels. It modulates the upregulation of miR-501-5p, which acts as a suppressor in LNM of HNSCC (60, 61).

#### 2.1.4 Other indicators

Essential biomarkers proposed for predicting LNM in EC include the overexpression of cortactin, MLL2, EIF4E, mTOR, and STC1 in ESCC, transcription of CD69, MyD88, and TCR4, and low expression of OLFM4 in EAC (62–64). A study has found that TSTA3 overexpression is associated with advanced LNM and was identified as a predictor in LNM of ESCC (65). Meanwhile, positive WTAP expression is correlated with advanced TNM stages in epithelial-mesenchymal transition (EMT) (66). In stage IIA ESCC after esophagectomy, FPXM1 overexpression predicts a higher 5-year recurrence of LNM (67). The elevated expression of MUC1 is a promising marker to predict advanced LNM and 5-year OS in ESCC after radical resection (68). In addition, the combined analysis of Annexin II, kindlin-2, and myosin-9 is more robust in predicting LNM in EC (69) (**Table 1**).

**Table 1:** Potential protein coding genes as indicators of LNM in EC.

Protein Coding Gene and Reference	Analysis Method and Samples	Expression or Mutation	Association between the LNM	Functions or Crosstalk
Vav1 (70)	IHC 112 ESCC cases	UP	Indicator Vav1-High 75.51% Vav1-Low 24.49% P=0.008	Activating actin cytoskeletal rearrangements and transcriptional alterations
TSTA3* (65)	IHC 104 ESCC cases	UP	Indicator TSTA3-High 35% TSTA3-Low 29% *X^2^ =* 4.876,P=0.043	NADP(H)-binding protein in glycosylation
TACC3 (71)	IHC 209 ESCC cases	UP	Indicator TACC3-High 59.78% TACC3-Low 40.22% P=0.028	A motor spindle protein in stabilization of the mitotic spindle and differentiation
KRT 15 (74)	IHC 128 EC cases	UP	Indicator KRT15 (pos. vs neg.) HR=3.011, 95%, P=0.002	The structural integrity of epithelial cells; Crosstalk with CATA4 and HER2
MACC1, ARG2, KAI1 (42)	IHC 106 HNSCC cases	UP UP DOWN	Indicator MACC1-High 89.19% MACC1-Low 10.81% P < 0.001 ARG2-High 56.76% ARG2-Low 43.24% P=0.002 KAI1-High 5.41% KAI1-Low 94.59% P < 0.001	MACC1: Participate in TRK, PIK3/Akt and HGF-MET signaling to modulate cellular growth, EMT, angiogenesis, cell motility, invasiveness and metastasis KAI1: downregulating in tumor and activated by p53
MACC1, Snail, KAI1 (43)	IHC 214 ESCC cases	UP UP DOWN	Indicator MACC1-High 74.03% MACC1-Low 25.97% P < 0.001 ARG2-High 83.12% ARG2-Low 16.88% P < 0.001 KAI1-High 16.88% KAI1-Low 83.12% P < 0.001	
WTAP* (66)	IHC 102 ESCC cases	UP	Predictor WTAP-High 77.35% WTAP-Low 22.64% Cor. Coefficient =0.275, P < 0.001	EMT signaling pathway; Tumor suppressor gene
SLC38A3 (93)	WGS and WES, CNA in the TCGA cohorts	DOWN	Indicator SLC38A3-High 41.8% SLC38A3-Low 58.2% P=0.026	Interact with SETDB1 to reduce Snail in the EMT pathway
RACN1.2 mRNA (94)	RT-qPCR, 96 ESCC cases	DOWN	Indicator RACN1.2 mRNA-High 26% RACN1.2 mRNA-Low.52% P=0.008	Tumor suppressor
FOXM1* (67)	Multivariate Cox regression analysis, 178 stage IIA ESCC cases	UP	Predictor-LMR FOXM1 (Low expression vs. Overexpression) HR=1.877, P=0.002	A transcriptional activator phosphorylated in M phase
TWIST1, MMP-21, HLAG-1 (49)	IHC, Cox multiple regression analysis 102 ESCC cases	UP	Indicator (Co-overexpression) TWIST1 and HLAG-1 Pos. = 0.037 TWIST and MMP-21 Neg. = 0.003	TWIST1: transcription factor promoting tumor cell invasion and metastasis, as well as metastatic recurrence; MMPs: the breakdown of extracellular matrix in normal physiological processes (embryonic development, reproduction, and tissue remodeling); CD44: a cell-surface glycoprotein involved in cell-cell interactions; INPP5A: mobilizing Intracellular calcium and acts as a second messenger CD147: immunoglobulin in spermatogenesis, embryo implantation, neural network formation, and tumor progression
TWIST1, MMP-13, CD44 (50)	RT-qPCR	UP	Indicator MMP-13-High 26% MMP-13-Low 13% P=0.042	
TWIST1, MMP-2, EGFR, INPP5A (51)	RT-qPCR, 58 ESCC cases	UP UP UP DOWN	Indicator INPP5A ↓/MMP-2 ↑ (Personal cor. = 0.807, Sig. = 0.000, P < 0.01) EGFR ↑/MMP-2 ↑ (Personal cor. = 0.416, Sig. = 0.048, P < 0.05)	
CD147, MMP-9 (52)	IHC, 78 type II/III AEG cases	UP	Indicator CD147-High/Low 28/10 (*X^2^ =* 9.119, P =0.003) MMP-9-High/Low 33/5 (*X^2^ =* 13.242, P < 0.001)	
ADAM17 (58)	RT-qPCR and SP, 50 ESCC cases	UP	Indicator ADAM17 1.172 ± 0.249, P < 0.001	membrane-anchored proteins involved in cell-cell and -matrix interactions
CLCA4 (61)	RT-qPCR and WB, 84 EC cases	DOWN	Indicator CLCA4-High 29.63% CLCA4-Low 70.37% P=0.048	Inhibition in cell viability, EMT, migration and invasion
CAPRIN1 (76)	RT-qPCR and IHC, 55 ESCC cases	UP	Diagnostic marker CAPRIN1-High 47.62% CAPRIN1-Low 38.10% P =0.031	Warburg effect and glycolysis by regulating METTL3 and WTAP
NNMT* (31)	scRNA-seq	UP	Indicator (Model) Metabolic: AUC=0.8391 Sen. = 0.2941, Spe. = 1 Integrated: AUC=0.872 Sen. = 0.7647, Spe. = 0.8824	M6A in the EMT signaling pathway by promoting nicotinate and nicotinamide metabolism
Annexin II*, kindlin-2*, myosin-9* (69)	IHC 147 ESCC cases	UP	Indicator Generation database 48.2% Validation database 56.5% X^2^ = 1.732, P=0.188	NA
PDIA3 ITGA5B1 (73)	IHC, 284 ESCC cases	DOWN	Indicator PDIA3-High 30.07% PDIA3-Low 69.93% P < 0.001 PDIA3-High 44.76% PDIA3-Low 55.24% P=0.001	PDIA3; interaction with lectin chaperones calreticulin and calnexin to modulate folding of newly synthesized glycoproteins
HOXB2* SEPT9* (34)	GWS the Illumina Infinium HumanMethylation450 BeadChip	DOWN UP	Predictor HOXB2-High 46.16% HOXB2-Low 53.85% P=0.0011 SEPT9-High 40.24% SEPT9-Low 36.54% P=0.0037	DNA Hypermethylation HOXD2 ↑ (P = 0.383) SETP9 ↑ (P = 0.095)
B3GNT3 (99)	IHC, 179 ESCC cases in GEO database	DOWN	Indicator P < 0.05	the synergy effect in the regulation of M2 macrophages

LNM, Lymph Node Metastasis; LMR, Lymphatic Metastatic recurrence; EC, Esophageal Carcinoma; ESCC, Esophageal squamous cell Carcinoma; AEG, Adenocarcinoma of Esophagogastric Junction; HR, Hazard Ratio; IHC, Immunohistochemistry; TMA, tissue microarray; SP, Streptavidin Peroxidase; MAGIC, The Medical Research Council Adjuvant Gastric Infusional Chemotherapy; UP, Upregulation; DOWN, Downregulation; AUC, Accuracy; Sen., Sensitivity; Spe., Specificity; Pos., Positive; Nge., Negative; Cor., Correlation; NA, Not applicable; GC-RiskAssigner, A seven-gene signature.

*The component is clearly stated as a predictive marker of LNM in EC in the studies.

Indicators: including two or more roles of predictor, diagnostic and prognostic markers. ↑, higher expression; ↓, lower expression.

Underlying markers (**Table 1**), for example, Vav1 (70), TACC3 (71), and HMGA2 (72), are positively associated with LNM of EC. Overexpressions of PDIA3 and ITGA5B1 are associated with advanced LNM and pTNM stages in a model for clinic risk stratification (73). KRT15 is overexpressed to promote malignant phenotypes in Barrett’s esophagus and EAC (74, 75). Caprin-1 overexpression indicates a poor prognosis in EC with LNM by regulating the Warburg effect and glycolysis (76–78). Overexpression of STMN1 (79) and NOLC1 (80) in ESCC indicates advanced LNM and poor prognosis by activating the PI3K/AKT pathway. PARK2 also promotes phosphorylation via the Hippo/YAP signaling pathway (81). Upregulation of Rad51 is involved in DNA repair and tumor metastasis (82). Overexpression of ATAD2 (83) and CDCA7 (84) activates TGF-β/Smad signaling to regulate LNM in ESCC, yet conflicting results are observed in RUNX3 (85). In another study, this tumor suppressor overexpressed but showed no significant association with the LNM of EC (86). Through the Wnt/β-catenin signaling pathway, RARα overexpression upregulates MMPs to promote ESCC metastasis (87). Besides, upregulation of FOXC2 and downregulation of ZNF750 are identified as metastatic and prognostic biomarkers, providing therapeutic targets for ESCC. ZNF750 dysfunction promotes tumorigenesis and metastatic ability of ESCC through the DANCR/miR-4707-3p/FOXC2 axis in a ceRNA manner (88). In ESCC, the TNFα/FOXC2/FA2H axis promotes lung metastasis by dysregulating ceramide metabolism via the NF-κB signaling pathways (89). Additionally, the coexpression of AGGF1 and FOXC2 functions in angiogenesis to promote the LNM in ESCC (90). The coexpression of PKM2 and HSP27 promoted the LNM of EC through ubiquitination (91). Additionally, PKM2 elevates STAT3 in TGF-β1-induced EMT to promote LNM (92). Some markers, including SLC38A3 (93), RCAN1.2 mRNA, and P21, are inversely associated with LNM and shorter survival (94, 95). The PTP family members suppress tumor migration and metastasis via RTK signaling pathways, and their silenced epigenetic modulation indicates malignancies (96). PTPRS and PTPRO deficiencies in ESCC are associated with aggressive LNM and other malignant phenotypes (97). PTPRO^low^/p-MET^high^ suppresses LNM of ESCC by dephosphorylating p-MET (98). B3GNT3 is suppressed and inversely associated with the LNM of ESCC through the synergistic effect on regulating M2 macrophages (99).

### 2.2 Noncoding RNAs

Long noncoding RNAs (lncRNAs) are potential predictive, diagnostic, and prognostic biomarkers in LNM of EC (**Table 2**). LINP1 and LOC440173 are potential indicators and activators of LNM in ESCC (100, 101). Two novel and tumor-specific lncRNAs, ENST00000508406.1 and NR_037652.1, facilitate the prediction of ESCC local invasion and LNM (102). Elevated SNHG6 in ESCC serves as an independent diagnostic biomarker for LNM, distant metastasis, and TNM stages (103). Furthermore, its silencing upregulates miR-186-5p and inhibits tumor migration by targeting HIF1α (104). Lnc-ABCA12-3 overexpression indicates an advanced TNM stage and an unfavorable prognosis, as it competitively binds to miR-200b-3p to upregulate FN1, which is involved in cell adhesion and migration. Moreover, exosome-mediated processes exacerbate metastatic progression in glycolysis through the TLR4/NF-κB signaling pathway (105, 106). LincIN is identified as a potential signature in BC metastasis (107). LincIN overexpression enhances the binding between NF90 and pri-miR-7 and downregulated mature miR-7, a tumor suppressor in various cancers, to elevate and indicate the LNM of ESCC (108, 109). SPRY4-IT1 is considered a diagnostic and predictive biomarker for ESCC surgical procedures and prognosis (110). As a sponge in many miRNAs (111), SPRY4-IT1 improves cellular viability and metastatic ability via ZNF703 overexpression (112) and TFG-β-induced EMT pathways, regulating LNM in ESCC (113). Additionally, lncRNA H19 and MEG promote LNM in EC via the STAT3/EZH2/β-catenin and PSAT1-dependent GSK-3β/Snail signaling pathways, respectively (114, 115). LINC00324 silences miR-493-5p to activate the MAPK1 signaling and enhance LNM in ESCC (116).

**Table 2:** Noncoding RNAs in the lymph node metastasis of esophageal cancer patients.

Biomarkers	Identified targets or signaling pathways	Function	Role	Reference
lncRNA SNHG6	NA	cell proliferation, migration, and invasion	(+)	(103)
	miR-186-5p/HIF1α			(104)
LincIN	NF90/miR-7/HOXB13			(108)
lnc-ABCA12-3	miR-200b-3p/FN1			(68)
	TLR/NF-κB	cell proliferation and glycolysis		(106)
SPRY4-IT1	ZNF703	cell proliferation, migration, and invasion and EMT-signaling		(112)
	TFG-β			(113)
LOC440173	miR-30d-5p/HDAC9			(101)
LncRNA H19	STAT3/EZH2/β-catenin			(114)
LINP1	NA			(100)
LINC00324	miR-493-5p/MAPK1		(–)	(116)
MEG	PSAT1-dependent GSK-3β/Snail			(115)
miR-10527-5p	Rab10/Wnt/β-catenin	cell proliferation, migration, and invasion and lymphangiogenesis	(+)	(121)
uc.189	EPHA2/P38MAPK/VEGF-C			(123)
miR-203*	KIF5C/Axin	cell proliferation, migration, and invasion	(–)	(124)
	miR-21			(126)
miR-133b	TAGLN2			(95)
miR-143-5p	Regulators of oncogenes (HN1, HMGA2 NETO2, STMN1, TCF3 and MET)			(128)
miR-143-3p	KRT80			
miR-625*	NA			(118)
miR-483-5p	NA		(+)	(117)
miR-320b	METTL3/m6A/miR-320b/ PDCD4/AKT in VEGF-C-independent manner	cell proliferation, migration, invasion, and EMT	(+)	(120)
miR19a-3p*	RAC1/CDC42-PAK1	tumor-specific miRNAs involved in local invasive and lymphatic metastasis	(+)	(122)
miR-17-3p* miR-18b-5p* miR19a-3p*	NA		(+)	(119)
ENST00000508406.1 NR_037652.1	dysregulation of lncRNA-messenger RNA pairs		(–)	(102)
miR-21*	miR-21/PDCD4 in apoptosis	tumor suppressor	(–)	(140)
miR-216a/b*	NA			(142)
miR-218*	NA			(141)
miR-20b-5p*	NA	oncogene	(+)	(143)

*The component is clearly stated as a predictive marker of LNM in EC in the studies. (+), positive expression; (-), negative expression; NA, not available.

MiRNAs are pivotal regulators of tumor growth and development. Expressions of miR-483-5p and miR-625 are positively and negatively correlated with LNM and OS, respectively, making them predictors of LNM and unfavorable prognosis in ESCC (117, 118). Xu Y et al. has demonstrated that miR-17-3p, miR19a-3p, and miR-18b-5p are tumor-specific miRNAs to regulate and predict the regional LNM of ESCC (119). Overexpression of miR-320b promotes the LNM of ESCC depending on m6A modification (120). Besides, exosome-mediated miR-10527-5p suppresses Rab10 by knocking down Wnt/β-catenin signaling to inhibit LNM and other malignant phenotypes of ESCC (121). Meanwhile, miR19a-3p upregulation and the uc.189-EPHA2 axis activates the RAC1/CDC42-PAK1 and the p38/MAPK/VEGF-C pathways to promote the formation of lymphatic vessels and the metastatic ability in ESCC (122, 123). In ESCC, exosomal uc.189 and miR-203 expressions are positively and negatively associated with metastasis and prognosis, respectively (124, 125). The malignancy is activated via the inhibition of KIF5C, the accumulation of Axin, and the sequential or synergistic regulation of miR-203 and miR-21 (124, 126). On the contrary, an anti-tumor miR-133b suppresses TAGLN2 and negatively impacts LNM (127). Additionally, miR-143-5p regulates oncogene expressions, including HMGA2, STMN1, and MET, thereby attenuating migration and invasion in EC cells, similar to the modulator of miR-143-3p in KRT80 (128). Moreover, miR-143-5p is observed to interact with numerous genes involved in LNM of EC, such as the negative regulator AGR2 in HNSCC (42).

### 2.3 Serum biomarkers

Serum markers have consistently attracted attention because of their sample availability. Traditional tumor markers in ESCC patients, such as CEA, CA125, CA199, CA724, and CA242, have been retrospectively analyzed. CA199 and CA125 are positively associated with LNM, and CA125 is a predominant predictor of LNM occurrence (129). Previous studies have demonstrated serum components’ predictive and prognostic role in LNM of EC, including TK1, CYFRA21-1, STMN1, PTEN, and TNFAIP8 (62, 130, 131). A novel homogeneous AlphaLISA improves the sensitivity (81%) and specificity (94%) for detecting serum STMN1, better indicating early-stage LNM in ESCC (132). Besides, the co-detection of autoantibodies against a panel of six tumor-associated antigens (p53, NY-ESO-1, MMP-7, Hsp70, PRDX6, and Bmi-1) is applied to predict early-stage LNM in ESCC (133). The lactate dehydrogenase to albumin ratio (LAR) demonstrates an association with advanced LNM and unfavorable prognosis in patients with resectable EC, regardless of NCRT (134, 135). Similar potential prognostic indices are utilized in CRC and other carcinomas (136, 137). Considering serum metabolites, amino acids are ideal metabolic biomarkers to predict LNM and monitor therapeutic efficacy (138). Combining three serum metabolites, valine, GABA, and pyrrole-2-carboxylic acid, enhances diagnostic efficacy in LNM of EC (139). Additionally, some novel miRNAs (**Table 2**) in serum, such as miR-21, miR-216a/b, and miR-218, play suppressive and predictive roles in LNM of EC (62, 140–142), while higher concentrations of miR-20b-5p detected in the serum are combined with a nomogram to predict advanced LNM of ESCC accurately (143). CircRNA secreted by ESCC cells, has-circ-0026611, is identified as a novel predictor of tumor prognosis (144). Meanwhile, blood cells may enhance a predictive and prognostic function in LNM of EC. Common blood components, such as platelets and neutrophils, promote the metastatic ability of cancers and interactions with tumor cells (145). Besides, some cell ratios, such as lymphocyte-monocyte, platelet-lymphocyte, and neutrophil-lymphocyte ratios (NLR), are positively associated with predicting and prognosticating LNM of ESCC. NLR is particularly effective in predicting LNM, indicating the need for thorough LND and consideration of preoperative adjuvant therapy for patients with clinical stage I ESCC (146). Meanwhile, NLR and other inflammatory markers, such as red cell width, leukocytosis, and neutrophilia, predict prognosis during NCRT, followed by esophagectomy (147–149). Moreover, the NLR/pre-albumin and NLR/albumin ratios demonstrate potential utility (150, 151).

## 3 Host-derived cells

### 3.1 Circulating tumor cells

Thanks to the development of the liquid biopsy, a novel noninvasive method to detect and evaluate changes in body fluids, the concentration of circulating tumor cells (CTCs) in EC, has emerged as a promising marker to assess tumor progression and monitor treatment efficacy (145, 152–154). Specific targets or changes in CTCs, such as a decrease in epithelial cell adhesion molecule (EpCAM) and the presence of cell surface vimentin, are linked to poor prognosis in patients with ESCC, underscoring their significance within the TME (155). Post-NCRT CTCs are applied to evaluate the degree of primary histopathological response and the necessity of esophagectomy (156). In addition, products expressed by CTCs might indicate therapeutic efficacy. For example, the sensitivity to radiotherapy is positively associated with mRNA expression of NRF2 and TP53 in CTCs; meanwhile, CD8+ T-cells accumulate in TME as NRF2 mRNA levels increase (157). Furthermore, CTCs are regarded as promising indicators in LNM of EC. However, this area has a paucity of research (158). The proportion of CTCs with chromosome 7 triploidy is related to distant metastasis and TNM stage (152). Higher levels of EpCAM and CEA in tissues are associated with LNM in EAC and could potentially be applied in tumor-target imaging for EAC (159). Another study has demonstrated that advanced LNM and TNM stages influenced the expression of PTP4A1 in ESCC and efficiently predicted the PTP4A1+ TCTs with an area under the ROC curve of 0.725 (160). Further studies are required to explore the association between CTCs and LNM in EC and other cancers.

### 3.2 Immune cell infiltration

Competitive interactions between tumor-infiltrating immune cells and cancer cells create unique lymph metastatic hubs and promote tumor occurrence, development, and metastasis (161). Previous research has demonstrated that innate immune cells in the TME, including tumor-infiltrating lymphocytes (TILs), tumor-associated macrophages (TAMs), and natural killer (NK) cells, play a key role in maintaining esophageal homeostasis and immune defense, providing potential targets for immunotherapy (162). In addition, immune checkpoint inhibitors, such as PD-1/PD-L1 inhibitors, have shown significant efficacy in various malignancies. In ESCC, researches have identified several biomarkers which may influence a patient’s response to immune checkpoint inhibitors (163, 164), yet few have focused on their roles in LNM of EC. For TILs, the silencing of T-cell factor/lymphoid enhancer factor lead to the downregulation of molecules such as SREBP1 (165) and G3BP1 (166) by phosphorylating different targets via Wnt/β-catenin signaling pathways in ESCC. Besides, the combined assessment of reduced CD8+/FOXP3+ TIL density and PD-L1 overexpression is proposed as a potential indicator for tumor differentiation and LN status in ESCC (167). In ESCC patients undergoing neoadjuvant chemotherapy, the PD-L1 status of tumor cells in positive LN tissues and the FOXP3/CD8 ratio in primary tumors are identified as prognostic factors for overall clinical outcomes (168). However, there is insufficient evidence regarding the impact of CD4+ TILs following NCRT for thoracic ESCC (169). The prevalence of PD-L1 immunoreactivity is significantly higher in IL-6-positive EC specimens, and T-cell functions against tumor cells, including proliferation and cytotoxicity, are inhibited (170). Therefore, PD-L1 determines radiation response and potentially serves as a prognostic indicator for patients with ESCC (171). The infiltration of activated cytotoxic T-cells is positively associated with PD-L1 in tumor tissues to predict 3-5-year OS and DFS, as well as TAMs (172). TAMs play a dual role in the TME, where TAM-1 has anti-tumor activity and TAM-2 promotes tumor metastasis. Studies have shown that the higher infiltration of TAM-2, especially CD68-TAMs, is associated with LNM and poor prognosis of EC (130, 173). A polarization tendency to TAM-2 is detected in EAC, and a higher ratio of M2/M1 macrophages serves as a sensitive marker to predict LNM and poor prognosis in EAC without NCRT (174). NK cells exhibit potent cytolytic activity against tumors, while dendritic cells are essential antigen-presenting cells to activate the immune response of TILs. Both cell types are critical regulators in the immune system and potential targets in the immunotherapy combined with PD-1/PD-L1. However, further research is necessary to develop novel markers and verify their efficacy in applying PD-1/PD-L1 inhibitors to target LNM of EC (163, 172).

## 4 Microbiota

Alterations in the microbiota have been linked to EC. Compared with direct access to esophageal samples, oral and intestinal flora could be used as noninvasive biomarkers to detect EC and have potential as practical assessment tools (175). In EC, microbiota have shown promise as tumor-associated biomarkers in precancerous lesions and early stages. Dysplasia is associated with bacterial alterations in EC, and Barrett’s esophagus, a precursor of EAC, is categorized into two main types based on Gram-positive or harmful bacterial components (176). Overall, the abundance and diversity of microbiota are lower in EC. Certain bacteria have been enriched or depleted in patients with EC compared to healthy controls, and the abundance of microorganisms is associated with OS and other clinicopathological characteristics (177). A consensus on the dominant bacteria in EC tissues is lacking. However, many studies have acknowledged the predominant position of *Fusobacteria* in the bacterial composition of EC associated with advanced tumor stages, identifying it as a potentially predictive and prognostic marker (178, 179). Meanwhile, a study has demonstrated the decreasing number of *Bacteroidetes*, *Fusobacteria*, and *Spirochaetes* (177). Another study has demonstrated that a shorter-term survivor has a higher number of *Lactobacillus* in tumors, while *Leptotrichia* is the opposite (180). There is an increasing abundance of *Prevotella* and a decreasing abundance of *Streptococcus* in the ESCC group, with different proportions in different stages (181). Meanwhile, brush samples from another analysis have revealed that the relative abundance of *Streptococcus* and *Prevotella* serve as significant defining characteristics among community types within the esophageal microbiota, and their combined abundance independently emerge as a prognostic predictor for individuals with ESCC (182). The degree of LNM alters microbiota distribution in ESCC (183), and the microbiota also potentially mediates LNM in EC. Besides a higher abundance of *Streptococcus* and *Prevotella* associated with the LNM group (182), it has been found that *Lactobacillus* (180) and *Fusobacteria* (184) are more abundant in tissues without LNM compared to those with LNM. Zhang et al. have confirmed the positively abundant *F. nucleatum* associated with LNM in ESCC and the depth of tumor infiltration (178).

Microbiota plays an essential role in tumor metastasis and other malignant phenotypes. Similar functions have been further investigated in other cancers, where metastatic activation occurs through the production of metabolites, bioactive compounds, and the modulation of inflammatory and anti-tumor responses. Genetic and epigenetic changes contribute to increased resistance to specific tumor treatments. Gut and intratumor microbiota can also reconstruct the distal organ microenvironment and form PMN so as to provide better living conditions for the transfer of tumor cells (180, 185). Chronic inflammation is known to be a precursor to EC and many other cancers. Some microbiota-derived metabolites, such as short-chain fatty acids, exert protective effects against inflammation and cancer, while others may promote carcinogenesis pathways (15). *Bacteroides* are closely associated with EC progression through increased inflammation and the activation of the TLR4/MyD88/NF−κB pathway triggered by lipopolysaccharide (LPS), a surface antigen. This underscores a strong connection between common LPS-induced signaling pathways in both inflammatory responses and the development and progression of EC (186). Induced by *Porphyromonas gingivalis* LPS *in vivo*, the NO secretion of macrophages in HNSCC is enhanced to proliferate and invade more aggressively (187). Alterations in microbiome diversity significantly modulate the immune system and impact immune surveillance and anti-tumor responses. The recognition of bacterial flagellin leads to increased nuclear localization of TLRs, especially TLR4 and TLR5. This upregulation is positively associated with malignant behaviors and metastatic abilities of Barrett’s esophagus and EAC (188, 189). When *Lactobacillus* plays a dominant role in intratumoral-bacterial compositions, it suppresses anti-tumor immune responses and facilitates the progression of EC (180). Robinson W et al. have developed a computational pipeline and suggested that myeloid cells in TME are important origins of microbiota. These cells contribute to inflammation and immunosuppressive responses in tumor tissues, since the abundance of myeloid cells increases with proinflammatory cytokines, IL-1β and CXCL8 upregulation (190). Based on the immune infiltration microenvironment, F. nucleatum invades and persists within different cells to enhance the LNM in ESCC. It facilitates cellular immune escape in Tregs (191). In ESCC cells, *F. nucleatum* activates the DNA damage response pathways and increases the secretion of cisplatin-induced senescence-associated secretory phenotype, significantly contributing to tumor progression and chemoresistance (178).

## 5 Discussion

EC represents an aggressive malignancy with significant implications for patient survival, particularly when it metastasizes. The lymphatic system is crucial for tumor cell dissemination. Considering thoracic EC, specific regional LNs are particularly susceptible to involvement, including the cervical paraesophageal, recurrent laryngeal nerve, subcarinal, those along the left gastric artery, lesser curvature, and paracardial LNs. A positive thoracic paraesophageal LN status indicates a more advanced disease (5). The length and complexity of the esophageal lymph network results in extensive drainage locations and unexpected “skip metastasis.” Differences in viewpoints and clinical practices regarding LNM in EC lead to disagreements in the locations of LNs between two major TNM staging systems. For example, in the 8th edition of the Union for International Cancer Control (UICC)/American Joint Committee on Cancer (AJCC), supraclavicular LNM is classified as M1 and celiac LNs are regional LNs. Meanwhile, the 12th edition of the Japan Esophageal Society (JES) categorizes a positive supraclavicular LN as M1a due to the possibility of a cure after dissection. However, celiac LNs are not regional for upper thoracic EC. The 2023 Guidelines of the Chinese Society of Clinical Oncology (CSCO) for EC follow the classification of the 8th edition of the UICC/AJCC (192). The incidence of LNM dramatically impacts treatments, including surgical interventions and applications of NCRT, as well as the prognosis and OS of patients.

The process of LNM in early EC is intricate and influenced by various factors, including tumor location, type, and depth of invasion. Each stage, from precancerous lesions to tumorigenesis to progression to PMN, involves distinct molecular changes driven by inflammatory, immune, and other responses (193). The risk of LNM increases as early EC invades deeper layers. Notably, when early EC invades the submucosal layer, the risk of LNM significant increases (3). Furthermore, the site of LNM is associated with the location and type of the tumor, with distinct patterns observed in upper, middle, and lower thoracic EC (194). Molecular markers have shown promise in predicting LNM and understanding the biological behavior of EC, informing individualized treatment. Despite the risk of LNM in early EC, not all patients develop it, as seen in patients diagnosed with clinical T1N0 EC who develop LNM postoperatively (195). Thus, comprehensive LN evaluation remains crucial, and further research is needed to explore the mechanisms, interactions, and determinants of tumor metastasis. Understanding the process and mechanism significantly improves the diagnostic accuracy, treatment outcomes, and prognostic management of patients with early EC. Management of early-stage EC differs by tumor stages. The pT1a stage can be managed endoscopically, yet additional interventions are required at the M3 stage. For the pT1b stage, surgical resection or NCRT yields better long-term outcomes (3). EC resection is considered the fundamental treatment for locally advanced cases. However, the 5-year postoperative OS differs significantly based on the presence of LNM. Patients with no LNM or only one LNM exhibit a 5-year OS rate exceeding 50%, while those with two or more LN metastases have a 5-year OS rate of less than 30% (8). A review of 3800 ESCC patients from the nationwide JES registry data suggested that LN dissection range should be based on the location of primary tumors (9). However, excessive LN dissection offers no additional advantages. Compared with standard two-field lymphatic dissection, total two-field lymphatic dissection failed to improve postoperative survival but caused more significant complications (10). Another randomized phase III trial reported that two- and three-field lymphatic dissection showed comparable rates and severities of postoperative complications in EC patients (196). In short, LN evaluation in EC therapy is essential in EC therapy.

Cancer biomarkers objectively measure and evaluate features that indicate normal and pathological processes or pharmacological responses to therapeutic interventions (17). Despite their potential, cancer biomarkers face significant challenges in clinical validation, particularly in detecting, diagnosing, and monitoring early-stage diseases to enhance long-term survival (16). It is estimated that approximately 50% of cancers are diagnosed at an advanced stage. Additionally, early biomarkers are often present in minuscule amounts, making it challenging to isolate accurate signals amidst normal human physiological noise. Potential risks, such as overdiagnosis and overtreatment, must also be addressed (197). Meanwhile, it is imperative to overcome scientific challenges to develop new biomarkers with enhanced sensitivity, specificity, and positive predictive value (198). Besides, combining traditional radiomics and biomarkers has demonstrated heightened predictive capabilities in developing and prognosis EC and other malignancies. This approach surpasses reliance on imaging modalities, such as EpCAM and CEA, novel targets used in tumor-target imaging approaches (159). Notably, signatures exhibit significant variations in radiomics-based imaging parameters. The diagnostic sensitivity and specificity of multislice CT, when coupled with the detection of CA19-9, Bcl-2, and CYFRA21-1, have exhibited superior performance in identifying LNM in EC patients compared to individual indicators (199). Increased Caprin-1 expression in EC samples is accurately anticipated through 18F-FDG PET/CT imaging, exhibiting a sensitivity of 70.8% and a specificity of 77.4%, suggesting the potential of Caprin1 as a robust prognostic biomarker (76). Likewise, elevated eIF6 expression is identified in EC utilizing 18F-FDG PET/CT, with an SUVmax threshold of 18.2, resulting in a predictive accuracy of 0.755 for tumor eIF6 expression (200).

**Figure 1:**
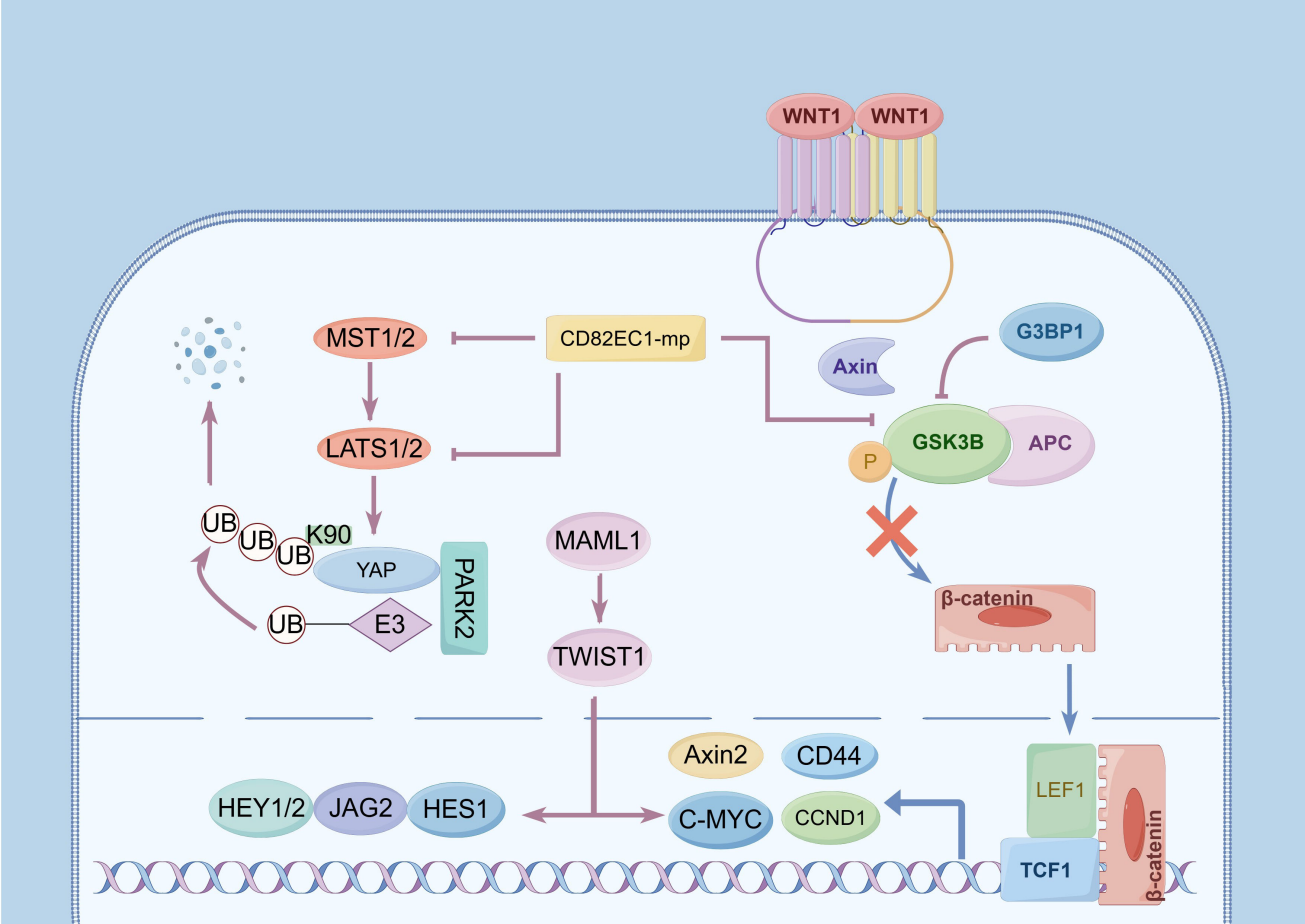
Activation of signaling pathways and upregulation of lymph node metastasis (LNM) of esophageal cancer (EC). (1) The Wnt/β-catenin signaling pathway: The phosphorylation of GSK3B significantly influences this pathway. G3BP1 promotes its phosphorylation, and further promotes the nuclear translocation of β-catenin, activates the expression of TCF/LEF, C-MYC, Axin2,CCND1, and other genes to participate in LNM. (2) The Hippo signaling pathway: MST1/2 and LAST1 are pivotal regulating targets, and YAP encodes a downstream nuclear effector in this pathway. Phosphorylation of MST1/2 and upregulation of LAST1 expression upregulate YAP phosphorylation and activate its proteasome-dependent degradation, and PARK2 facilitates YAP ubiquitination at site K90. CD82EC1-mP can inhibit the above two pathways to inhibit LNM by suppressing the phosphorylation of GSK3β and MIST1. (3) The Notch signaling pathway: MAML1 is an essential transcriptional co-activator in this pathway, and its overexpression induces TWIST1 expression, upregulating downstream targets including C-MYC, JAG2, HEY1, HEY2, and HES1.

**Figure 2:**
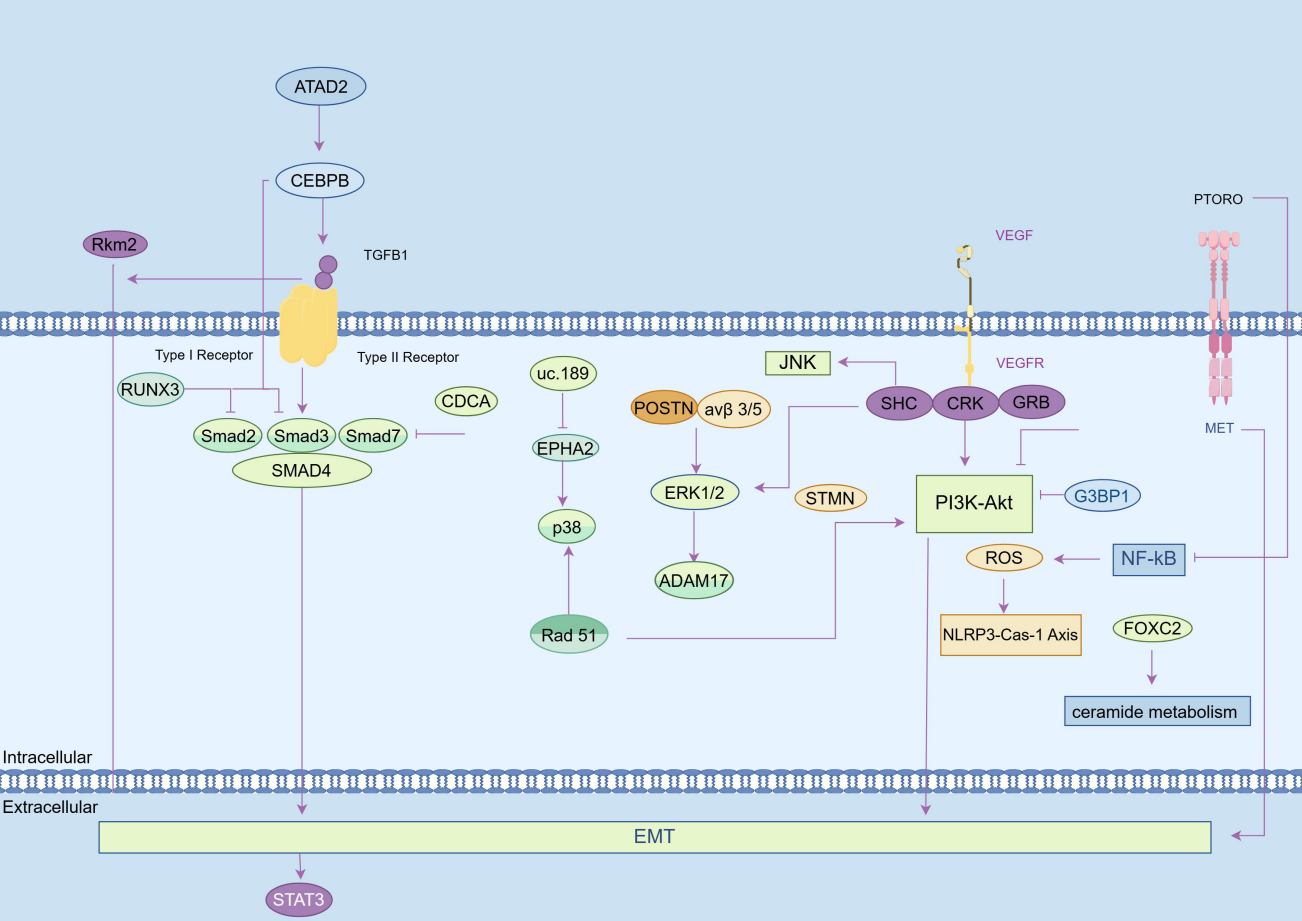
EMT-dependent mechanisms in promoting the lymph node metastasis (LNM) of esophageal cancer (EC). (1) NF-κB signaling pathway: Increased FOXC2 dysregulates ceramide metabolism to activate this pathway and elevate the LNM of EC. Conversely, PTPROt deficiency reduces ROS, inhibiting the NLRP3-Caspase-1 axis and suppressing LNM. (2) RTK signaling pathway: VEGF/VEGFR induces dimerization, phosphorylation, and activation of PTKs, to recruit adaptor proteins such as CRK, SHC, GRB2, thereby promoting LNM of EC. (3) PI3K/AKT signaling pathway: NOLC1 overexpression activates the PI3K/AKT pathway to enhance LNM in EC. G3BP1 knockdown inhibits this pathway by reducing p-PI3K and p-AKT expression levels. Inhibition of the PI3K pathway also suppresses STMN1 expression. Rad51 overexpression upregulates p38 phosphorylation and Snail levels to elevate Akt phosphorylation. (4) MAPK signaling pathway: uc.189 inhibits EPHA2 expression to activate p38 and ultimately promote LNM; POSTN binding to integrin αvβ3/αvβ5 increases ERK1/2 phosphorylation and upregulates ADAM17, leading to tumor aggressiveness. (5) JAK-STAT pathway and TGF-β pathway: Activation of these pathways impedes LNM. PKM2 promotes STAT3 phosphorylation through TGF-β1-induced EMT. ATAD2 interacts with C/EBPβ for nuclear translocation and binding for TGF-β1 promoter activation. TGF-β1 promotes Smad3 phosphorylation and interaction with Smad4 for regulation within the nucleus. Increased RUNX3 reduces Smad2/Smad3 phosphorylation, suppressing TGF-β1-induced EMT. CDCA7 overexpression promotes Smad4 but inhibits Smad7 expression to facilitate EMT in ESCC.

In precision oncology, gene expression signatures related to immunotherapy have shown promise as markers. Analyzing gene expression patterns of immune cells in the TME can provide insights into how tumors evade immune surveillance and guide personalized treatment strategies. Some studies have proposed that genetic evolution is a driving force for tumor metastasis, suggesting that carcinomas might be associated with groups of genes sharing similar characteristics rather than operating independently. Several models with varying sensitivity and specificity levels have been developed for EC, such as ferroptosis (201) and mitochondrial (202) genes. Despite extensive sequencing, there is limited evidence of metastasis-specific genomic alterations, and the underlying mechanisms are still unknown (145). In addition to gene expression products, there are increasingly specific markers. Glycans on the cell surface are structure-variable and tissue-specific, which play a role in tumor occurrence and development. When combined with Lens culinaris lectin, they serve as a predominant predictor in the early detection of LNM in ESCC (203). TMNs significantly modulate tumor development through immune infiltrates and other aspects of tumor progression. Even before metastasis initiation, the primary tumor selectively influences the PMN (204) to enhance the colonization of CTCs (205). Regarding TME and PNM, PD-L1 expression and the abundance and activity of CD8+ T-cells play crucial roles in indicating anti-tumor responses and predicting the efficacy of targeted and immunotherapy (170). The presence of PMN is pivotal for tumor metastasis, as the local microenvironment alone cannot support the dissemination and colonization of CTCs in distant organs and tissues (206). Significant alterations in the microenvironment of LNs and vessels occur before metastasis, creating a PMN that is conducive to the growth of CTCs (207). The formation of this niche is currently debated, with several key factors identified, including changes in the LN vasculature, influence on lymphatic endothelial cells, modulation of the stiffness of the ECM, involvement of fibroblastic reticular cells, and creation of an immunosuppressive microenvironment (4). EVs can establish PMNs and support rapid tumor dissemination to transport cargoes such as nucleic acids and proteins, influencing various signaling pathways, suppressing defensive immune reactions, polarizing tumor-promoting phenotypes, expressing the PD-1 checkpoint protein (208), and targeting EVs represents a promising therapeutic strategy (207). Research and application of these biomarkers demonstrate their potential to enhance the efficacy of cancer treatment and improve patients’ quality of life, particularly in immunotherapy and other targeted therapies. Signaling pathways that regulate LNM in EC are various, such as Wnt/β-catenin, MAPK, and RTK; some rely on EMT signatures (**Figures 1**, **2**). Future studies should focus on underlying therapeutic targets and novel treatments for clinical application.

The relationship between the microbiota and various diseases has attracted significant attention. Gut microbiota are involved in body homeostasis and influenced by many factors, including diet, smoking, drinking, obesity, and intake of drugs, whose imbalance leads to various diseases (209). Through blood and lymph circulations, gut microbiota reach even distant organs and tissues, where they find and settle in suitable environment, including the TME, becoming one of the essential origins of intratumor microbiota, which are significant components of the cancerous ecosystem (210). Recent studies have demonstrated the existence of intratumor microbiota in several cancers (21, 170), which could increase tumor metastasis by regulating the intrinsic characteristics of tumor cells via EMT regulation, as well as extrinsic characteristics through vessel barrier blocking and modifications in the PMN (211). In BC, intratumor microbiota emerges as a novel and promising biomarker for diagnosis, prognosis, and therapeutic assessments (212–214), prompting further exploration of its role in EC and other cancers. Thanks to next-generation sequencing and non-invasive detection of oral and fecal samples, it is more convenient and practical to explore the pathogenesis and diagnostic or prognostic applications of microbiota in esophageal diseases (215, 216). Studies have suggested that alterations in the gut microbiota composition could be associated with an increased risk of esophageal disorders, such as eosinophilic esophagitis, gastroesophageal reflux disease, and achalasia (217). Recent research on EC has suggested that microbiome may influence its occurrence, development, and early diagnosis or prognosis. Moreover, research is ongoing to explore new avenues for clinical treatments and management, and manipulating the gut microbiome could provide new avenues for clinical treatments and management and enhance the efficacies of chemotherapy and immunotherapy (180, 190). Forsythoside A rejuvenates the diversity of microorganisms to show its efficacy against ESCC and significant implications for clinical implementation (218), and probiotics or dietary modifications aimed at restoring a healthy gut microbiome could complement conventional cancer treatments in ECA (219). The use of antibiotics in EC should be carefully evaluated, as certain species like *F. nucleatum* could provide a novel targeting strategy to optimize clinical outcomes of cisplatin resistance (178). However, more rigorous research is required to confirm the connection between bacterial biomarkers and LNM in EC, and to translate findings into clinical practice. Current evidence fails to support the view that microbiota is an indicator of EC, particularly EAC. Furthermore, detailed investigations are required to determine whether noninvasive oral or fecal specimens can provide detection as sensitive and specific as biopsy, or even better (175).

Despite ongoing research into biomarkers for evaluating LNM in EC, there is a lack of FDA-approved tests and other commercially available options. A cost-benefit analysis by European surgeons indicated that, among current biomarkers for gastrointestinal cancers, CEA outperformed CA19-9 and CA125 in lower financial cost, higher sensitivity, and better diagnostic accuracy for metastases at presentation (220). Advances in molecular research and clinical trials may eventually lead to reliable biomarkers for routine clinical use in EC management. Our review presents a comprehensive analysis of recent research on potential biomarkers and their underlying mechanisms in LNM of EC. We aim to provide new insights and advancements in predictive approaches by identifying unique biomarker signatures. Utilizing these biomarkers can improve pre- and post-surgical assessments, leading to the development of personalized and effective therapeutic strategies for better patient outcomes and quality of life. Furthermore, the identified biomarkers and their associated pathways may serve as targets for therapeutic interventions in EC patients. However, there is limited research on the specific mechanisms and pathways of biomarkers in LNM among patients with EC. Most biomarkers referring to LNM of EC are prognostic, involving mutations and gene expressions. Only a few, such as serum biomarkers and CTCs, have shown the potential to predict or monitor therapeutic outcomes. In conclusion, future research efforts should concentrate on these areas to enhance our understanding and improve clinical practices.
